# Investigation of the pathophysiology of cardiopulmonary bypass using rodent extracorporeal life support model

**DOI:** 10.1186/s12872-017-0558-6

**Published:** 2017-05-15

**Authors:** Ru-Wen Chang, Chien-Ming Luo, Hsi-Yu Yu, Yih-Sharng Chen, Chih-Hsien Wang

**Affiliations:** 1 Department of Physiology, College of Medicine, National Taiwan University, No. 1, Sec. 1, Ren’ai Rd., Zhongzheng Dist., Taipei, 10051 Taiwan; 2 Department of Surgery, National Taiwan University Hospital, Hsin-Chu Branch, NO. 25, Lane 442, Sec. 1, Jingguo Rd., Hsin-Chu, 30059 Taiwan; 3 Cardiovascular Surgery, Department of Surgery, National Taiwan University Hospital, No. 7, Zhongshan S. Rd., Zhongzheng Dist., Taipei, 10002 Taiwan

**Keywords:** Asphyxial cardiac arrest, Extracorporeal life support, Inflammatory response, Rat model

## Abstract

**Background:**

Extracorporeal life support (ECLS) systems are life-saving devices used for treating patients with severe cardiopulmonary failure. In this study, we implemented a rat model of ECLS without the administration of inotropes or vasopressors.

**Methods:**

The rats underwent 5 min of untreated asphyxial cardiac arrest and were resuscitated by ECLS for 30 min. The right external jugular vein and right femoral artery were separately cannulated to the ECLS outflow and inflow, respectively. Thereafter, ECLS was terminated, wounds were closed, and mechanical ventilation was provided for another 90 min. Subsequently, blood gas and hemodynamic analyses were performed. The plasma levels of C-reactive protein (CRP), interleukin (IL)-6, IL-10, and tumor necrosis factor-alpha (TNF-α) were measured 120 min after reperfusion.

**Results:**

The metabolic rate of lactate in the group of asphyxial cardiac arrest rescued by ECLS was slow; therefore, the pH at 120 min after reperfusion was significantly lower in this group than that in the group of normal rats treated with ECLS. The hemodynamic data showed no between-group differences. The plasma levels of CRP, IL-6, IL-10, and TNF-α increased after ECLS treatment.

**Conclusions:**

We successfully established a rodent ECLS model, which might be a useful approach for studying the pathophysiology induced by ECLS under clinical conditions.

**Electronic supplementary material:**

The online version of this article (doi:10.1186/s12872-017-0558-6) contains supplementary material, which is available to authorized users.

## Background

Extracorporeal life support (ECLS) systems preserve adequate blood flow and oxygen supply in both adults [1, 2] and children [3, 4], and they have been described as life-saving devices. Because the components, such as a membrane oxygenator and catheters [5], and driving force of ECLS systems are nonbiological and nonphysiological, their effects on patients have not been completely elucidated. Studies are needed to elucidate the nonbiological and nonphysiological effects of ECLS. Although ECLS models in large animals, such as porcine models [6, 7], are well established because of the equipment, small animal models still have the potential for research in resuscitation studies. Rodent models are more economical when a high number of experiments must be performed. Furthermore, they provide various molecular tools such as genetic knockout or relevant diseases.

For the present study, we designed a rodent ECLS model for the following purposes: 1) to identify whether ECLS can be performed without a blood transfusion or the administration of drugs such as inotropes or vasopressors and by performing only crystalloid priming throughout the experiment; and 2) to measure the plasma levels of C-reactive protein (CRP), interleukin (IL)-6, IL-10, and tumor necrosis factor-α (TNF-α) 120 min after reperfusion.

## Methods

### Animals

Male Wistar–Kyoto rats (450–550 g) were randomly divided into a group of normal rats that did not receive ECLS (sham; *n* = 11), a group of normal rats treated with ECLS (NC + ECLS; *n* = 11) and a group of asphyxial cardiac arrest rats rescued by ECLS (CA + ECLS; *n* = 11). Two rats were housed per cage, and they had free access to Purina chow and water under a 12-h light–dark cycle. The experiments were conducted in accordance with the Guide for the Care and Use of Laboratory Animals, and the study protocol was approved by the Animal Care and Use Committee of National Taiwan University (IACUC number: 20140024).

### Anesthesia and surgical preparation

Sodium pentobarbital (Sigma Chemical Co., St. Louis, MO, USA; 50 mg kg^−1^) was administered intraperitoneally to anesthetize the rats, and intravenous reinjections (35 mg kg^−1^) were performed every hour. The surgical sites (neck and right and left groin) were shaved, and the lungs were ventilated using a ventilator (Model 131, New England Medical Instruments, Medway, MA, USA). Ventilation was performed with room air through a 14G plastic catheter (B. Braun Medical, Bethlehem, PA, USA) at a tidal volume of 8 mL kg^−1^ and respiratory rate of 70 breaths per min. The rats were placed in the supine position and a rectal temperature probe (TP-K01 and TES-1300, TES Electrical Electronic Corp., Neihu Dist., Taipei, Taiwan) was inserted for the continuous monitoring of rectal temperature. Rectal temperature was maintained at 36 °C by using a circulating warm water blanket (B401H, Firstek Scientific Co. Ltd., Xinzhuang Dist, New Taipei City, Taiwan; TP22G, Gaymar Industries, Inc., Orchard Park, NY, 14,127 USA) and a heating lamp. The electrocardiogram (ECG) of lead II was recorded using a Gould ECG/Biotech amplifier (Gould Electronics, Cleveland, OH, USA).

The surgical sites were shaved and disinfected with betadine. The left femoral artery was cannulated using a Millar catheter (model SPC 320, size 2F; Millar Instrument, Houston, TX, USA) for the continuous monitoring of arterial pressure, and the left femoral vein was cannulated using PE-50 tubing for drug administration throughout the experiment. A 20G IV catheter (B. Braun Medical, Bethlehem, PA, USA) was inserted into the right femoral artery for ECLS inflow and arterial blood sampling. Cannulation of the right external jugular vein for ECLS venous outflow was then performed using a customized template-modified 5-hole 14G catheter, which was advanced to the junction of the right atrium and superior vena cava. This catheter was heparin-locked with 500 UI of heparin to prolong the activated clotting time to longer than 300 s. The arterial blood gas, arterial lactate, and hematocrit (i-STAT CG-4+ and 6+ cartridge, Abbott Point of Care, Princeton, NJ, USA) were determined at the baseline. The same procedure was used for all groups.

### Cardiac arrest and ECLS

After instrumentation and the collection of the baseline parameters, a neuromuscular blockade was performed through the intravenous administration of pencuronium bromide (Sigma Chemical Co., St. Louis, MO, USA; 1 mg kg^−1^) for 5 min. Subsequently, the ventilator was switched off for 5 min to induce asphyxial cardiac arrest. Circulatory arrest was defined as a mean arterial blood pressure (MAP) of <20 mmHg because this pressure is commensurate with the cessation of all tissue blood flow.

**Fig. 1:**
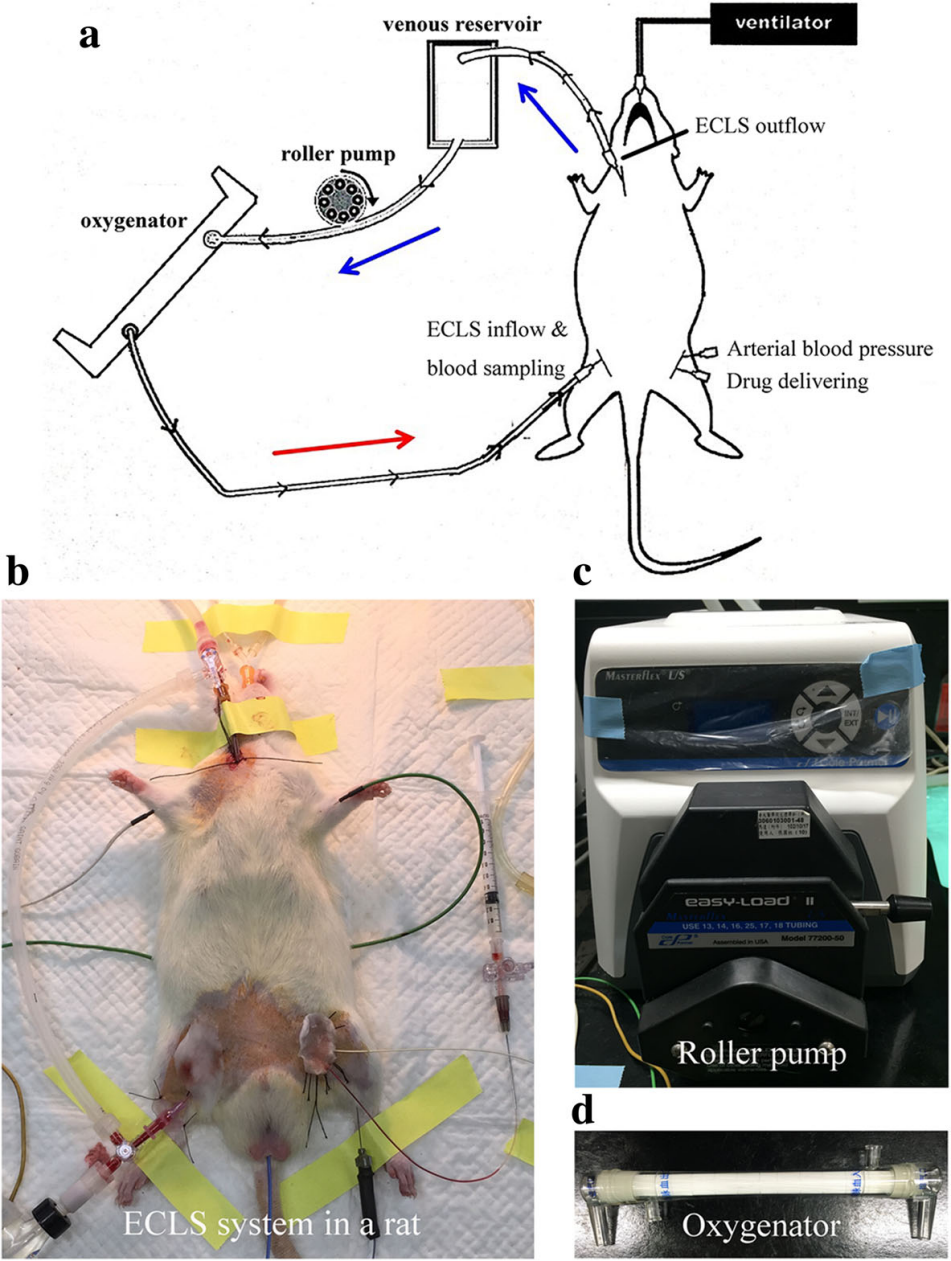
Rodent extracorporeal life support (ECLS) system. (**a**) schema of the rodent ECLS model; (**b**) demonstration of our rodent ECLS model; (**c**) roller pump; and (**d**) oxygenator

The ECLS circuit (Fig. 1) designed for the rats consisted of an open venous reservoir (TERUMO®, Tokyo, Japan; 5-mL syringe), a membrane oxygenator (Micro-1 Rat Oxygenator, Dongguan Kewei Medical Instrument Co., Ltd., Guangdong, China), a heat exchanger (Radnoti Glass Technology Inc., Monrovia, CA, USA), silicone tubing (ID 1.6 mm), and a roller pump (Masterflex, Barrington, IL, USA) that was primed using 19–20 mL of Plasma-Lyte A (Baxter, Deerfield, IL, USA). After 5 min of asphyxia, reperfusion was initiated by starting the ECLS system, which was continued for 30 min.

### Discontinuation of ECLS and intensive care unit phase

After 30 min of ECLS, the rats were weaned off the system by removing the right jugular catheter within 5 min, and the incision was closed in two layers by using an absorbable suture for the subcutaneous layer and skin. Arterial samples for blood gas analysis, hematocrit, and lactate were collected at 30, 60, 90, and 120 min after the commencement of reperfusion, defined as the start of ECLS. We did not perform a blood transfusion or administer drugs such as bicarbonate, inotropes, and vasopressors. The pump was primed only with crystalloid throughout the experiment. All rats were humanely euthanized at the end of the experiment.

### Estimation of inflammatory response

To estimate the inflammatory response, plasma levels of CRP (Immunology Consultants Laboratory Inc., Portland, OR, USA), IL-6 (BioLegend Inc., San Diego, CA, USA), TNF-α (BioLegend Inc., San Diego, CA, USA), and IL-10 (Abcam, Cambridge, UK) were measured using enzyme-linked immunosorbent assay kits.

### Statistical analysis

All data are reported as the mean ± standard error of the mean (SEM). The Mann–Whitney *U* test was used to compare differences between the groups at the same time points. Kaplan–Meier curves were plotted to depict the survival trend. The log-rank test was used to compare the risk between the two groups. In the mortality cases, the survival time was defined as the duration from the baseline to death; among the survivors, the duration was from the baseline to 120 min after the start of reperfusion. Statistical significance was defined as *P* < 0.05.

## Results

**Fig. 2:**
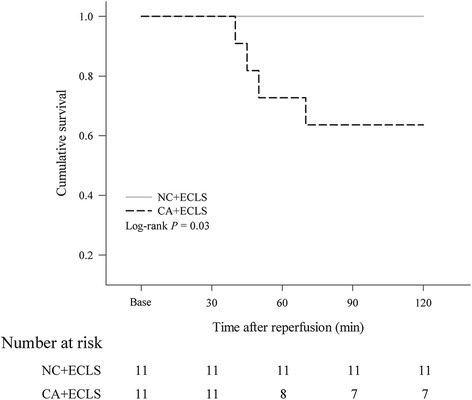
Kaplan–Meier plot of the survival curves in the NC + ECLS and CA + ECLS groups. CA + ECLS: asphyxial cardiac arrest rats rescued by ECLS; ECLS: extracorporeal life support; NC + ECLS: normal rats treated with ECLS. A significant difference (*P* = 0.03) was observed in the overall survival rate between the CA + ECLS and NC + ECLS groups

The Kaplan–Meier curves in Fig. 2 represent the cumulative survival of rats after reperfusion in the NC + ECLS and CA + ECLS groups. Figure 2 shows that not all rats were successfully resuscitated in the CA + ECLS group; therefore, the data for this group depend on the number of rats that survived until 30, 60, 90, or 120 min after reperfusion. Furthermore, the overall survival differed significantly between these two groups (*P* = 0.03).

**Fig. 3:**
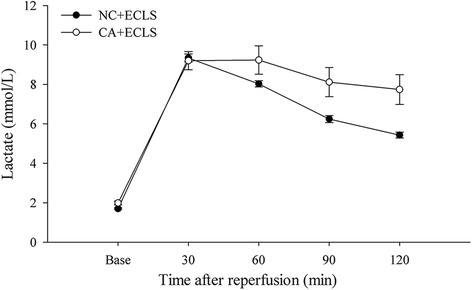
Lactate level, which is used to describe tissue perfusion, gradually decreased throughout the experiments. The line diagram (mean ± SEM) represents the whole blood lactate levels in both groups. The data shown depended on the number of rats surviving at each time point. CA + ECLS: asphyxial cardiac arrest rats rescued by ECLS; ECLS: extracorporeal life support; NC + ECLS: normal rats treated with ECLS. No significant differences were observed between the CA + ECLS and NC + ECLS groups at the same time points

Surgical preparation time and baseline parameters, namely animal body weight, arterial blood gas, hematocrit, and hemodynamic variables, did not differ between the NC + ECLS and CA + ECLS groups (Tables 1 and 2). The changes in arterial blood gas values after reperfusion during the experiments are presented in Table 2. The ion variable measurements before and after ECLS in the NC + ECLS and CA + ECLS groups are presented in the additional file (Additional file 1: Table S1). At 30 min after reperfusion, the hematocrit decreased to approximately half of the baseline value because the rats in both groups did not receive a blood transfusion during ECLS treatment. After the rats were weaned off the ECLS system, the retained solution in the circuit was reinfused into the rats in the intensive care unit phase; therefore, the hematocrit value gradually increased throughout the experiment. Simultaneously, the increase in MAP after reperfusion also depended on the reinfusion of the retained solution and not on the effects of inotrope or vasopressor administration. The lactate level, which reflects tissue perfusion, decreased in both groups but the decreasing rate was considerably more rapid in the NC + ECLS group (Fig. 3). All the basic parameters in the sham group (*n* = 2) are showed in the additional file (Additional file 2: Table S2).

**Table 1:** Comparison of baseline properties between groups

	NC + ECLS (*n* = 11)	CA + ECLS (*n* = 11)	*P-*value
Body weight (g)	472±4	467±5	0.8237
Duration of preparation (min)	103±1	102±1	0.7847

Variables are presented as mean ± SEM. CA + ECLS: asphyxial cardiac arrest rats rescued by ECLS; ECLS: extracorporeal life support; NC + ECLS: normal rats treated with ECLS

**Table 2:** Arterial blood gas, hematocrit, and hemodynamic variables before and after ECLS

	Baseline	30 min	60 min	90 min	120 min
pHa					
NC + ECLS	7.54±0.01	7.44±0.01	7.38±0.01	7.40±0.01	7.42±0.01
CA + ECLS	7.55±0.01	7.43±0.02	7.33±0.02	7.33±0.02	7.32±0.02^*^
PaO_2_ (mmHg)					
NC + ECLS	92±2	120±3	99±1	89±1	83±1
CA + ECLS	90±1	114±3	102±3	87±4	84±4
PaCO_2_ (mmHg)					
NC + ECLS	28.3±0.6	18.5±0.3	21.8±0.4	23.6±0.4	24.5±0.5
CA + ECLS	25.9±0.7	19.0±0.8	21.9±1.3	28.6±1.8	27.5±1.9
Base excess (mmol L^-1^)					
NC + ECLS	1.5±0.2	−11.4±0.3	−12.3±0.3	−10.4±0.3	−8.5±0.3
CA + ECLS	0±0.2	−11.5±0.7	−13.8±1.2	−10.1±1.3	-10.7±1.5
Hematocrit (%)					
NC + ECLS	40±0	19±0	21±0	23±0	24±0
CA + ECLS	40±0	17±0	21±0	23±1	25±0
Heart rate (beats min^−1^)					
NC + ECLS	381.8±3.7	348.5±3.8	398.6±2.7	413.4±2.4	429.6±2.2
CA + ECLS	384.9±4.4	367.1±5.1	417.2±3.7	430.6±4.8	419.3±8.9
Mean arterial pressure (mmHg)					
NC + ECLS	121.1±0.9	41.0±0.8	58.9±1.2	72.8±1.2	75.1±1.0
CA + ECLS	113.3±1.3	35.5±1.9	55.6±2.5	73.1±3.0	76.4±3.3

Variables are presented as mean ± SEM. The data depended on the number of surviving rats at each time point. CA + ECLS: asphyxial cardiac arrest rats rescued by ECLS; ECLS: extracorporeal life support; NC + ECLS: normal rats treated with ECLS.

*: *P* < 0.05 when the CA + ECLS group was compared with the NC + ECLS group at the same time point

**Fig. 4:**
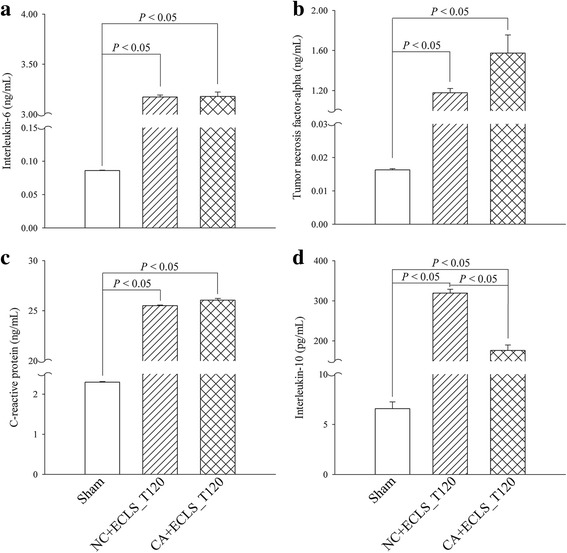
ECLS treatment increased the plasma levels of pro-inflammatory and anti-inflammatory cytokines compared with the sham group. The pro-inflammatory cytokines were (**a**) interleukin-6 (IL-6), (**b**) tumor necrosis factor-α (TNF-α), and (**c**) C-reactive protein (CRP); (**d**) the anti-inflammatory cytokine was interleukin-10 (IL-10). Bar diagrams (mean ± SEM) depict summarized data. Data represent *n* = 11 in the sham and NC + ECLS groups and *n* = 7 in the CA + ECLS group. CA + ECLS: asphyxial cardiac arrest rats rescued by ECLS; ECLS: extracorporeal life support; NC + ECLS: normal rats treated with ECLS; Sham: normal rats that did not receive ECLS treatment; T120: 120 min after reperfusion

Comparing the NC + ECLS and CA + ECLS groups at 120 min after reperfusion, the plasma levels of pro-inflammatory cytokines (i.e., IL-6, TNF-α, and CRP) and the anti-inflammatory cytokine (i.e., IL-10) were all significantly lower in the sham group than those in the NC + ECLS and CA + ECLS groups (Fig. 4). There showed no significant differences in IL-6, TNF-α, and CRP levels between NC + ECLS and CA + ECLS groups (Figs. 4a–c); however, the IL-10 levels differed significantly between these two groups (Fig. 4d).

## Discussion

In this study, we demonstrated that asphyxial cardiac arrest resuscitated using ECLS in rats was reproducible and enabled the survival of even the rats that were weaned off ECLS. However, the survival rate in the CA + ECLS group was only 63.6%, which was significantly lower than that in the NC + ECLS group. The mechanical-ventilation settings were similar to those in a previous study [8]; the only difference was the inspired gas. Because we used room air instead of higher inspired oxygen fraction as the inspired gas, the levels of blood gases (PaO_2_ and PaCO_2_) in our study were lower than those reported in previous studies [9–12]. However, the blood gas levels in the CA + ECLS and NC + ECLS groups did not exhibit any differences at the same time points in our study.

The rate of the decrease in lactate in the CA + ECLS group was lower than that in the NC + ECLS group. This might have caused the higher mortality rate and lower pH observed in the CA + ECLS group. The inability to metabolize lactate properly, which could cause acidosis in the body, might be a reason for the mortality rate in the CA + ECLS group [13]. Therefore, the adjustment of the acid–base balance appears necessary.

The MAP levels in both groups exhibited their lowest value at 30 min after reperfusion and gradually increased during the remainder of the experiments. Ali et al. used phenylephrine (an α_1_ agonist) to augment MAP during the ECLS reperfusion [14] so did in others [15]. By contrast, we used the remaining blood in the ECLS circuit rather than drugs to increase MAP in our study. This not only improves MAP but also could increase hematocrit levels.

In the CA + ECLS and NC + ECLS groups, hematocrit levels were significantly lower after reperfusion than at the baseline, because a blood transfusion was not performed during or after ECLS. Despite the decrease in hematocrit caused by hemodilution, sufficient oxygenation was maintained in both groups. Animal models have been reported to maintain adequate oxygen delivery to satisfy the whole body’s oxygen demands without blood product administration when the ventilator was supplied with higher inspired oxygen fraction [10, 12, 16–18]. However, we ventilated only with room air even in the absence of a blood transfusion to maintain acceptable oxygenation.

The results indicated that, compared with the sham group, the plasma levels of the inflammatory cytokines (i.e., IL-6, TNF-α, and CRP) and anti-inflammatory cytokine (i.e., IL-10) were significantly higher in the NC + ECLS and CA + ECLS groups after treatment with ECLS. In the previous study, Fujii et al. demonstrated that the inflammatory responses getting aggravated during ECLS perfusion [8]. Herein, we suggested that a systemic inflammatory response continued to occur in our model even after the extracorporeal circulation was removed. Although the contact of blood with artificial surfaces [19], the nonpulsatile flow caused by the pump [20], and the nonlaminar flow during extracorporeal circulation [21] are possible factors responsible for the inflammatory response during ECLS, they cannot explain the inflammatory response sustained even after the rats were weaned off ECLS.

Extracorporeal circulation has been reported to disrupt the tight junctions of the small intestinal epithelial cells and cause gut barrier dysfunction. Then, because of this gut barrier dysfunction, bacteria in the intestinal lumen might translocate into the blood and increase the blood level of endotoxins such as lipopolysaccharide [22, 23]. This bacterial translocation might explain the inflammatory response even after the ECLS was disconnected in our study. In addition, the bacteria can not only induce an inflammatory response but also activate an anti-inflammatory response through stimulation of the vagus nerve [24]. This might explaining the higher level of IL-10 in both ECLS treated groups compared with sham group.

The current model contains some still limitations. Because rodent blood volume is approximately 7% of body weight [25], it is difficult to measure the time dependent performances of the pro-inflammatory and anti-inflammatory cytokines after the rats are weaned off ECLS. The priming volume of our ECLS circuit was approximately 19–20 mL, which accounts for 61% of the blood volume of the rats. However, this was the minimum volume that we could attain in our model. Because we could not miniaturize the circuit, rats are the smallest model that we can operate with ECLS. Moreover, ECLS treatment time in clinical settings is considerably longer than that in our study, and the complex comorbidities seen in patients are difficult to mimic.

## Conclusions

In this study, we successfully developed a novel and reproducible miniature rodent ECLS model without blood transfusion or drug administration. We also introduced asphyxial cardiac arrest to our ECLS model and resuscitated the rats successfully. However, the adjustment of the acid–base balance appears necessary, particularly in rats with induced asphyxial cardiac arrest. The proposed model might provide opportunities for studying the mechanism of the pathophysiology of cardiopulmonary bypass without drug effects, even in mutant rats.

## Additional files

Additional file 1: Table S1. Ion variable measurements before and after ECLS. (DOCX 64 kb)

Additional file 2: Table S2. Body weight, arterial blood gas, hematocrit, and hemodynamic variables showed in sham group (*n* = 2). (DOCX 71 kb)

## Abbreviations

CRP: C-reactive protein
ECLS: Extracorporeal life support
IL: interleukin
MAP: Mean arterial blood pressure
TNF-α: Tumor necrosis factor-alpha
