# Supplementary material for: Investigation of the pathophysiology of cardiopulmonary bypass using rodent extracorporeal life support model
Source: BMC Cardiovasc Disord. 2017 May 15;17:123. doi: 10.1186/s12872-017-0558-6 (PMC5433070; doi:10.1186/s12872-017-0558-6)
Supplement: Supplementary file 1 — Ion variable measurements before and after ECLS. (DOCX 64 kb) [file 12872_2017_558_MOESM1_ESM.docx]

| Table S1  Ion variable measurements before and after ECLS. | | | | | | | | | |
| --- | --- | --- | --- | --- | --- | --- | --- | --- | --- |
|  | Baseline | 30 min | | | 60 min | 90 min | | | 120 min |
| Na^+^ (mmol/l) |  | |  |  | | |  |  | |
| NC+ECLS | 139.5$\pm$0.1 | 136.8$\pm$0.2 | | | 139.3$\pm$0.2 | 141.5$\pm$0.2 | | | 142.2$\pm$0.2 |
| CA+ECLS | 140.5$\pm$0.2 | 137.7$\pm$0.7 | | | 140.4$\pm$0.4 | 141.3$\pm$0.7 | | | 143.3$\pm$0.4 |
| K^+^ (mmol/l) |  | |  |  | | |  |  | |
| NC+ECLS | 4.1$\pm$0.0 | | 3.8$\pm$0.3 | 4.1$\pm$0.0 | | | 4.2$\pm$0.0 | 4.5$\pm$0.1 | |
| CA+ECLS | 4.0$\pm$0.0 | | 4.2$\pm$0.1 | 4.2$\pm$0.2 | | | 4.4$\pm$0.2 | 4.8$\pm$0.3 | |
| Cl^-^ (mmol/l) |  | |  |  | | |  |  | |
| NC+ECLS | 101.9$\pm$0.1 | | 99.2$\pm$0.3 | 101.5$\pm$0.2 | | | 103.0$\pm$0.2 | 104.5$\pm$0.2 | |
| CA+ECLS | 104.1$\pm$0.3* | | 102.7$\pm$0.6* | 102.4$\pm$0.4 | | | 102.9$\pm$0.4 | 104.0$\pm$0.4 | |

Variables are presented as the mean $\pm$ SEM. The data depended on the number of surviving rats at each time point. CA+ECLS: asphyxial cardiac arrest rats rescued by ECLS; ECLS: extracorporeal life support; NC+ECLS: normal rats treated with ECLS.

* *P* < 0.05 when the CA+ECLS group was compared with the NC+ECLS group at the same time point.
