# Supplementary material for: Investigation of the pathophysiology of cardiopulmonary bypass using rodent extracorporeal life support model
Source: BMC Cardiovasc Disord. 2017 May 15;17:123. doi: 10.1186/s12872-017-0558-6 (PMC5433070; doi:10.1186/s12872-017-0558-6)
Supplement: Supplementary file 2 — Body weight, arterial blood gas, hematocrit, and hemodynamic variables showed in sham group (n = 2). (DOCX 71 kb) [file 12872_2017_558_MOESM2_ESM.docx]

| Table S2  Body weight, arterial blood gas, hematocrit, and hemodynamic variables showed in sham group (n=2). | | | | | | | | | |
| --- | --- | --- | --- | --- | --- | --- | --- | --- | --- |
|  | Baseline | 30 min | | | 60 min | 90 min | | | 120 min |
| pHa |  | |  |  | | |  |  | |
| Sham | 7.57$\pm$0.00 | 7.60$\pm$0.02 | | | 7.55$\pm$0.01 | 7.62$\pm$0.02 | | | 7.63$\pm$0.03 |
| PaO_2_ (mmHg) |  | |  |  | | |  |  | |
| Sham | 98$\pm$8 | | 99$\pm$8 | 96$\pm$1 | | | 91$\pm$0 | 101$\pm$1 | |
| PaCO_2_ (mmHg) |  | |  |  | | |  |  | |
| Sham | 27.8$\pm$0.2 | | 23.3$\pm$0.5 | 26.7$\pm$1.4 | | | 23.2$\pm$0.7 | 22.3$\pm$0.2 | |
| Base excess (mmol/L) |  | |  |  | | |  |  | |
| Sham | 3.5$\pm$0.4 | | 1.5$\pm$1.8 | 1.0$\pm$2.1 | | | 2.5$\pm$1.2 | 2.5$\pm$1.8 | |
| Lactate (mmol/l) |  | |  |  | | |  |  | |
| Sham | 1.97$\pm$0.37 | | 2.06$\pm$0.34 | 2.33$\pm$0.16 | | | 3.04$\pm$0.12 | 2.60$\pm$0.59 | |
| Na^+^ (mmol/l) |  | |  |  | | |  |  | |
| Sham | 141.0$\pm$0.0 | | 139.0$\pm$0.0 | 139.5$\pm$1.1 | | | 139.5$\pm$0.4 | 139.0$\pm$0.7 | |
| K^+^ (mmol/l) |  | |  |  | | |  |  | |
| Sham | 4.0$\pm$0.1 | | 4.2$\pm$0.2 | 4.0$\pm$0.1 | | | 3.5$\pm$0.2 | 4.0$\pm$0.1 | |
| Cl^-^ (mmol/l) |  | |  |  | | |  |  | |
| Sham | 104.5$\pm$0.4 | | 105.0$\pm$0.7 | 104.5$\pm$0.4 | | | 104.0$\pm$0.0 | 104.5$\pm$0.4 | |
| Hematocrit (%) |  | |  |  | | |  |  | |
| Sham | 44$\pm$0 | | 44$\pm$0 | 43$\pm$1 | | | 42$\pm$2 | 43$\pm$0 | |
| Heart rate (beats min^-1^) |  | |  |  | | |  |  | |
| Sham | 378.6$\pm$23.4 | | 397.2$\pm$15.0 | 376.5$\pm$1.7 | | | 400.7$\pm$12.4 | 388.6$\pm$13.0 | |
| Mean arterial pressure (mmHg) |  | |  |  | | |  |  | |
| Sham | 106.8$\pm$1.6 | | 106.9$\pm$2.8 | 102.9$\pm$0.0 | | | 109.0$\pm$2.2 | 118.8$\pm$7.2 | |

Variables are presented as the mean $\pm$ SEM. The data depended on the number of surviving rats at each time point.
